# A machine learning approach to small area estimation: predicting the health, housing and well-being of the population of Netherlands

**DOI:** 10.1186/s12942-022-00304-5

**Published:** 2022-06-06

**Authors:** Markus Viljanen, Lotta Meijerink, Laurens Zwakhals, Jan van de Kassteele

**Affiliations:** National Institute for Public Health and the Environment - RIVM, PO Box 1, 3720BA Bilthoven, Netherlands

**Keywords:** Small area estimation, Machine learning, Extreme gradient boosting, Health and welfare

## Abstract

**Background:**

Local policymakers require information about public health, housing and well-being at small geographical areas. A municipality can for example use this information to organize targeted activities with the aim of improving the well-being of their residents. Surveys are often used to gather data, but many neighborhoods can have only few or even zero respondents. In that case, estimating the status of the local population directly from survey responses is prone to be unreliable.

**Methods:**

Small Area Estimation (SAE) is a technique to provide estimates at small geographical levels with only few or even zero respondents. In classical individual-level SAE, a complex statistical regression model is fitted to the survey responses by using auxiliary administrative data for the population as predictors, the missing responses are then predicted and aggregated to the desired geographical level. In this paper we compare gradient boosted trees (XGBoost), a well-known machine learning technique, to a structured additive regression model (STAR) designed for the specific problem of estimating public health and well-being in the whole population of the Netherlands.

**Results:**

We compare the accuracy and performance of these models using out-of-sample predictions with five-fold Cross Validation (5CV). We do this for three data sets of different sample sizes and outcome types. Compared to the STAR model, gradient boosted trees are able to improve both the accuracy of the predictions and the total time taken to get these predictions. Even though the models appear quite similar in overall accuracy, the small area predictions at neighborhood level sometimes differ significantly. It may therefore make sense to pursue slightly more accurate models for better predictions into small areas. However, one of the biggest benefits is that XGBoost does not require prior knowledge or model specification. Data preparation and modelling is much easier, since the method automatically handles missing data, non-linear responses, interactions and accounts for spatial correlation structures.

**Conclusions:**

In this paper we provide new nationwide estimates of health, housing and well-being indicators at neighborhood level in the Netherlands, see ’Online materials’. We demonstrate that machine learning provides a good alternative to complex statistical regression modelling for small area estimation in terms of accuracy, robustness, speed and data preparation. These results can be used to make appropriate policy decisions at a local level and make recommendations about which estimation methods are beneficial in terms of accuracy, time and budget constraints.

## Introduction

The health and housing situation of the Dutch population is regularly monitored by local authorities. In the Netherlands an extensive national survey, called the “Health Monitor Adults and Elderly (Gezondheidsmonitor Volwassenen en Ouderen)” (HeMo), is designed to gather the health information at a national and municipality scale. The survey is carried out every four years by the 25 Dutch municipal Health services (MHS) [1] and is coordinated by the National Institute for Public Health and the Environment (RIVM) and Statistics Netherlands (CBS). A second survey, called the “Housing Survey of Netherlands (Woon Onderzoek Nederland)” (WoON), collects information about the current and desired living situation. This survey is carried out every three years by Statistics Netherlands (CBS) in collaboration with the Ministry of Internal Affairs (BZK) to assess the living situation of the Dutch population and their wishes and needs in the field of housing [2]. Even though both surveys include tens to hundreds of thousands of respondents, many of the Netherland’s 13,808 neighborhoods have only few or even zero respondents. In that case, directly estimating the health and welfare at neighborhoods level from the survey responses becomes impossible.

To resolve this challenge, the field of Small Area Estimation (SAE) has developed many methods, we refer to the literature for a comprehensive review of their strengths and weaknesses [3]. Regression approaches train a model on the observed responses predicted by an auxiliary data set of features of the respondents, such as age and educational level. In our problem, regression models are particularly suitable because Statistics Netherlands has an extensive administrative data set of demographic variables covering the entire Dutch population. We can therefore train a regression model based on the observed health-related indicators or living quality ratings using the sub-population of respondents in the survey. We then use this model to predict the missing survey responses of the remaining population. We can aggregate these predictions to any desired geographical level, such as municipalities, districts and neighborhoods.

A previous study has shown that complex regression models, such as a structured additive regression (STAR) model, achieve well-calibrated predictions of several health-related indicators at neighborhood level in the Netherlands [4]. The statistical regression-based approach has however some drawbacks. First, one has to select a model. This model should handle non-linear relations between the auxiliary data and the outcome, but also interactions and spatial effects. This usually requires a statistician who translates prior knowledge into a model, and careful data preparation. Second, and this applies to any model, the model should be trained and over-fitting should be prevented by regularization techniques, such as shrinkage or penalization methods [5]. Third, such complex statistical models may run into computational difficulties when handing huge data sets with millions of records.

Machine learning techniques can be used as an attractive alternative. They have many potential benefits compared to statistical models: more accurate predictions, faster training times for large data sets, more robustness to different data sets, and they require less work and knowledge from the statistician to design and implement. Machine learning is gaining recognition as a potential solution to the problem of SAE, even though it has seen limited use so far [6–10]. In our study, we apply machine learning to a massive prediction problem with an important spatial component: we generate predictions for every adult individual in the Netherlands. From a machine learning perspective, the above procedure is a supervised learning task. A supervised machine learning method can be seen as a general learning algorithm that takes any data set of features with examples of correct labels, and outputs a model that is able to predict unknown labels from the features.

In this paper we provide estimates of health, housing, and welfare indicators at municipality, district and neighborhood level using gradient boosted trees (XGBoost), a well-known machine learning technique [11]. We compare the performance of XGBoost to a complex statistical structured additive regression model (STAR), designed specifically for this problem [4]. We show that it is possible to predict the indicators at the individual level using XGBoost and that the results are generally better compared to complex statistical SAE models.

## Methods

### Data sources

In this section we introduce the data sets that are being used in this study. Dutch policy makers are interested in small area estimation of many different surveys. It is important to verify that machine learning is able to adapt to each of these surveys, which can have quite different characteristics. The method could then be used in the future to automatically produce the desired estimates. We consider three surveys:

1. “Health Monitor Adults and Elderly” (HeMo)
2. “Housing Research of the Netherlands” (WoON)
3. “Experienced noise disturbance from traffic” (Noise) subsurvey of HeMo.

For the survey data sets we construct a corresponding population data set with features based on administrative data provided by Statistics Netherlands. The population data set includes the entire Dutch population, aged 18 years or older for the year of the survey, with the exception of institutionalized people who are not included in either survey. A secured identification number that links each survey to the administrative data was assigned to each respondent. Authorization for this linkage was provided by CBS. Disclosure and tracing of individuals is not possible. The data sets are summarized in Table 1. The outcomes and features will be described in further detail in the next sections.

**Table 1:** Data sets used in this study

Survey	Year	Respondents	Outcomes	Type	Population	Features
HeMo	2020	539,895	34	Binary	13,845,474	14
WoON	2018	67,523	8	Continuous	13,510,237	22
Noise	2016	202,065	10	Binary	10,366,070	21

#### Health monitor—HeMo

The Adult and Elderly Health Monitor (HeMo) is an extensive national survey about self-reported health and well-being. The survey is administered every four years by the MHS regions in collaboration of the National Institute for Public Health and the Environment (RIVM) and Statistics Netherlands (CBS). At the time of writing, data has been collected for years 2012, 2016, and 2020. In this paper we consider the results of the 2020 survey. Data were collected in September 2020 on 539,895 respondents (3.9% of the Dutch adult population) by an online questionnaire [1]. We consider 34 binary health indicators chosen from the survey. These include indicators like alcohol use, smoking behaviour, body weight, physical and mental health, disabilities, financial difficulties, exercise, loneliness, self perceived health and informal care giving. Table 9 in the Appendix describes all of the indicators.

#### Housing research of the Netherlands—WoON

The Dutch housing survey (WoON) is also a national survey, administered every three years by Statistics Netherlands (CBS) in collaboration with the Ministry of Internal Affairs (BZK). The survey gathers information about the current and desired housing situation of non-institutionalized Dutch residents aged 18 years or older. At the time of writing, data has been collected for years 2006–2018. Here we consider the results of the 2018 survey. Data were collected between August 2017 and April 2018 on 67,523 respondents (0.54% of the Dutch adult population, non-institutional) by an online questionnaire [2]. We consider eight continuous ratings of housing satisfaction from the survey. The first seven ratings are 1–5 scores (1: strongly disagree, 2: disagree,..., 5: strongly agree) and the last rating is a composite score between 1 and 10. Table 10 in the Appendix gives a full description.

#### Experienced noise disturbance from traffic—noise

Noise disturbance from traffic is an important subset of questions in the 2016 HeMo survey. The source of noise is identified as road, train or air traffic noise. The road noise is further divided into any noise, noise from traffic less than 50 km/h, or noise from traffic more than 50 km/h. The noise nuisance is then classified as (1) serious (2) moderate or serious, resulting in 10 indicators in Table 11 in the Appendix. The administrative data is expected to provide only limited information about how much the Dutch population experiences noise disturbance from traffic. Instead, the measured noise level at the individual’s spatial location can be expected to provide most information. RIVM has developed a noise dispersion model that predicts the noise level at each address based on actual measurements, knowledge of road and rail infrastructure, flight paths, etc. [12]. For this task, we only use the 18–64 year old population and add this as an additional feature to the administrative data. Table 12 in the Appendix describes the noise level predictions.

#### Administrative data for the population

The features of the Dutch population are obtained from CBS administrative data. Based on previous research and expert opinion of MHS and RIVM, we used 14 features for modelling the responses for HeMo, WoON, and Noise. At individual level we use age, sex, ethnicity, marital status, and highest completed level of education. At household level we have household type, size, income source, home ownership, income and assets and X- and Y-coordinates of the home address. At neighborhood level we have the address density. For WoON, we also add eight additional neighbourhood features to test how well the models developed for HeMo generalize. First, the percentage of uninhabited houses, single-family homes, non-rental houses, social housing, and houses built before 2000. Second, the distance from an individual’s house to the nearest forest, backwater and public green. For Noise, we include the additional noise level predictions from the RIVM noise dispersion model.

**Table 2:** Summary of features used for prediction in HeMo and WoON

Feature	Categories/median (min.–max.)	% missing
Age	50 (18–108)	0.0
Sex	Male	0.0
	Female	
Ethnicity	Netherlands	0.0
	Morocco	
	Turkey	
	Suriname	
	Netherlands Antilles	
	Other non-western	
	Other western	
Marital status	Single	0.6
	Married	
	Divorced	
	Widowed	
Education (descriptions Table 13)	Basis	37.8
	VMBObk	
	VMBOgt	
	MBO23	
	MBO4	
	HAVO-VWO	
	HBO-WO-BAC	
	HBO-WO-M/PhD	
Household type	Single person household	0.0
	Unmarried without children	
	Married without children	
	Unmarried with children	
	Married with children	
	Single parent family	
	Other	
Household size	2 (1–10)	0.0
Household income source	Wage	2.4
	Wage director/shareholder	
	Self-employed	
	Unemployment benefit	
	Social assistance benefit	
	Disability benefit	
	Old-age pension	
	Other benefit	
	Student loan	
	Property income	
Home ownership	Homeowner	2.0
	Rental no allowance	
	Rental with allowance	
Household income (percentile)	63 (1–100)	2.4
Household assets (percentile)	56 (1–100)	2.4
Neighborhood address density	71 (1–100)	0.0
*x*-coordinate (m)	140,348 (13,666–277,711)	0.0
*y*-coordinate (m)	453,926 (306,922–611,538)	0.0

**Table 3:** Additional features used for prediction in WoON

Feature	Median (min.–max.)	% missing
% in the neighborhood of		
Uninhabited houses	3 (0–100)	0.2
Single-family houses	78 (0–100)	0.2
Owner-occupied houses	62 (0–100)	0.2
Social rental houses	26 (0–100)	0.2
Houses built before 2000	91 (0–100)	0.2
Distance (m) from home to closest		
Forest	1463 (0–16,271)	0.0
Backwater	2080 (0–24,852)	0.0
Public green	280 (0–10,139)	0.0

Tables 2 and 3 summarize these features. For categorical features, the categories are given. For continuous features, the median and range are given. The percentage of missing features in the population data is provided as well. The most significant problem is the missing highest completed level of education. However, educational level is a very important predictor for health because it can be used to distinguish students that differ from other young individuals with low income. Therefore this feature is considered too important to be excluded. The handling of missing feature data will discussed in the next sections. To obtain demographic and spatial features of every individual as close to survey date as possible, we use a reference date of September 1, 2020 for HeMo. For WoON we have access to the dates on which people filled in the survey and therefore use these dates to obtain demographic and spatial information about the household on this exact date. However, several data sources are only updated yearly, so for those we use the reference date of January 1, 2018.

#### Municipalities, districts and neighborhoods

In 2020, the CBS administrative data had individuals registered in 25 municipal health regions, 355 municipalities, 3163 districts and 13,478 neighborhoods. Municipal health services work through a common system for several municipalities in a given region, called an MHS region, to carry out a number of tasks in the field of public health. Municipalities are administrative divisions that have corporate status and powers of self-government or jurisdiction. Their duties are delegated to them by the central government. Districts are nested within municipalities and neighborhoods within districts. Districts and neighborhoods are coherent regions that typically share similar population characteristics such as age, social structure, economic area, geographical features, etc. They have no formal status; they are defined for administrative purposes and data collection by CBS.

### Models

#### Formalization of the prediction problem

We train a model based on the observed health indicators, living quality ratings, or noise disturbance indicators using the sub-population of respondents in the survey. We then use this model to predict the missing survey responses of the remaining population and generate predictions for every adult individual in the Netherlands. From a machine learning perspective, this is a supervised learning task.

Suppose we have a set of *N* individuals denoted by $[N]=\{1,2,\ldots N\}$. The survey is a subset ${\mathcal {I}}\subset [N]$ of *n* individuals from this population. From the administrative data set we get a vector of *d* features $x_i\in {\mathbb {R}}^d$ for each individual *i*. From the survey data set we get responses $y_i\in \{0,1\}$ (classification) or $y_i\in {\mathbb {R}}$ (regression) if the individual was in the survey ($i\in {\mathcal {I}}$) and $y_i=\text {NA}$ otherwise ($i\notin {\mathcal {I}}$).

The goal of supervised learning is to learn an unknown function $f:{\mathbb {R}}^d \rightarrow \{0,1\}$ or $f:{\mathbb {R}}^d \rightarrow {\mathbb {R}}$ from a set of training examples ${\mathcal {D}}=\{(x_i,y_i)\}_{i\in {\mathcal {I}}}$ each consisting of an input vector $x_i\in {\mathbb {R}}^d$ and an associated output which may be binary $y_i\in \{0,1\}$ or real-valued $y_i\in {\mathbb {R}}$. This function should approximate the unknown true function $y_i\approx f(x_i)$ on the training data $i\in {\mathcal {I}}$ with the aim of generalizing to new data $i\notin {\mathcal {I}}$ which is not seen in the training phase. When predicting the prevalence or average rating for policy makers, we would use the observed responses when available in the survey and the model predictions for every individual not in the survey. We therefore define the predicted response $y^*_i$ as:

$$\begin{aligned} y^*_i :={\left\{ \begin{array}{ll} y_i &{} i\in {\mathcal {I}} \\ f(x_i) &{} i\notin {\mathcal {I}} \end{array}\right. } \end{aligned}$$

Given a partition of individuals into $r=1,\ldots ,K$ mutually exclusive geographic regions ${\mathcal {R}}_r$, where ${\mathcal {R}}_1\cup \ldots \cup {\mathcal {R}}_K = [N]$ and ${\mathcal {R}}_r\cap {\mathcal {R}}_s=\emptyset \text { if }r\ne s$, we calculate the predicted prevalence or average rating in each region as simply the average:

$$\begin{aligned} p_r = \frac{1}{\Vert {\mathcal {R}}_r\Vert }\sum _{i\in {\mathcal {R}}_r} y^*_i \end{aligned}$$

Model agnostic prediction intervals can be determined as follows. The goal is to calculate $b=1,\ldots ,B$ bootstrapped statistics $p^{(b)}_r$ for the true mean in each region, and take their 95% percentile intervals as prediction intervals. To quantify model uncertainty, we bootstrap resample the training data as data sets $\{{\mathcal {D}}^{(b)}\}_{b=1}^{B}$ and denote a model trained in each as $f^{(b)}$. The outcome uncertainty in classification is a Bernoulli trial $y_i \sim \text {Bern}(p_i)$ from true probability $p_i$ and we assume the outcome in regression follows a normal distribution $y_i \sim {\mathcal {N}}(\mu _i,\sigma _i^2)$ given true mean $\mu _i$ and variance $\sigma _i^2$. The outcomes are independent given their true means. We estimate the mean with the bootstrapped model prediction and a constant variance $\sigma ^2 = \frac{1}{|{\mathcal {I}}|}\sum _{i\in {\mathcal {I}}}(y_i - {\overline{f}}(x_i))^2$ where ${\overline{f}}(x_i)=\frac{1}{B}\sum _{b=1}^{B} f^{(b)}(x_i)$. For every bootstrap sample $b=1,\ldots ,B$, we train model $f^{(b)}$ and sample the outcomes. For unknown outcomes ($i\notin {\mathcal {I}}$) we simulate $y^{(b)}_i \sim \text {Bern}(f^{(b)}(x_i))$ in classification and $y^{(b)}_i \sim {\mathcal {N}}(f^{(b)}(x_i),\sigma ^2)$ in regression. The known outcomes ($i\in {\mathcal {I}}$) are taken as observed $y^{(b)}_i := y_i$, which corresponds to a finite sample correction. The bootstrapped mean is then calculated as $p^{(b)}_r= \frac{1}{\Vert {\mathcal {R}}_r\Vert }\sum _{i\in {\mathcal {R}}_r} y^{(b)}_i$. Their 95% percentile intervals are the prediction intervals.

#### Null model

The first model is the null model. This model simply predicts the mean in the Netherlands: either prevalence or the average rating. Each individual and therefore each geographical region gets the same outcome:

$$\begin{aligned} f(x_i) = \frac{1}{\Vert {\mathcal {I}}\Vert }\sum _{j\in {\mathcal {I}}} y_j \end{aligned}$$

#### Structured additive regression model (STAR)

The second model under consideration is a statistical model specifically designed for small area estimation in the Netherlands. This model is a structured additive regression model (STAR) and provides an elaborate framework for modelling nonlinear effects using penalized B-splines and spatial information using Markov random fields. The generalized linear model and the generalized additive model can be considered as a special case of the STAR model [5].

In this paper we use an updated version of the STAR model that was presented by [4]. The current STAR model used by the RIVM has several improvements. First, the updated model includes educational level. Second, more two-way interactions are included: age by sex, age by ethnicity, age by marital status, age by educational level, sex by ethnicity, sex by marital status and sex by educational level. Third, all features enter the model using basis functions (B-splines for continuous features, dummies for categorical features) and penalization of the regression coefficients. This also enables automated feature selection, i.e., features that are not relevant will not be selected in the model, resulting in a more parsimonious model [13]. The original STAR model was used to make the HeMo 2012 predictions, but the updated model was used for the HeMo 2016 predictions. It will also be used as a reference for the HeMo 2020 predictions.

Estimation of (hyper)parameters is carried out with restricted maximum likelihood (REML) using the bam function in the mgcv R package [14, 15]. Because of the size of the data set and the complexity of the model, it is impossible to fit this model to the entire data sets. The data sets is therefore split by MHS regions and a separate model was fit to each split, as in the original paper [4]. To avoid boundary effects, the model for each MHS region also includes all data within a 10 km buffer around the considered MHS region. These models have identical specification but the estimated coefficients and smoothing penalty may differ between regions.

Missing feature values are not allowed in the STAR model. We therefore used the random forest algorithm to sequentially impute the missing values from the least missing feature to the most missing feature.

#### Gradient boosting (XGBoost)

The third model is a gradient boosting with decision trees. This is a general machine learning technique for classification, regression, etc. It has been shown across many data sets that sophisticated machine learning models (random forest, kernel methods, neural networks, boosting with decision trees) tend to have good classification accuracy in tabular data sets such as our problem [16]. We chose gradient boosting with decision trees because previous studies that have compared machine learning methods in SAE have found them to work slightly better [8, 9] and they are computationally much faster in our large data sets.

Boosting creates a strong prediction model iteratively as an ensemble of weak prediction models, where at each iteration a new weak prediction model is added to compensate the errors made by the existing weak prediction models. Gradient boosting generalizes other boosting methods by allowing the optimization of arbitrary differentiable loss functions. Typically decision trees are used as the model, which is called gradient boosted trees. A decision tree model called Classification And Regression Trees (CART) can be used for both classification and regression. Given an arbitrary loss function $L(y_i, f(x_i))$, the gradient boosted trees can be described in a general form as [13, 17]:

1. Start with a constant function: $f_0(x) = \text {argmin}_{\gamma _0}\sum _{i=1}^{n}L(y_i,\gamma _0)$
2. For each iteration $t = 1,\ldots ,T$ construct a new tree:
  - For example $i = 1,\ldots ,n$, compute the negative gradient
    $$\begin{aligned} r_{it} = - \left[ \frac{\delta L(y_i, f(x_i)]}{\delta f(x_i)}\right] _{f=f_{t-1}} \end{aligned}$$
  - Fit a classification and regression tree to the targets $r_{i,t}$ giving terminal nodes $j=1,\ldots ,J_t$ with corresponding terminal regions $R_{j,t}$, i.e. the set of examples in terminal node *j* at iteration *t*.
  - For $j=1,\ldots ,J_t$ compute the terminal node estimates
    $$\begin{aligned} \gamma _{j,t} = \text {argmin}_{\gamma } \sum _{x_i \in R_{j,t}} L(y_i, f_{t-1}(x_i) + \gamma ) \end{aligned}$$
  - Using learning rate $\alpha$ update a new function $f_{t}(x)$ as
    $$\begin{aligned} f_{t}(x) = f_{t-1}(x) + \alpha \sum _{j=1}^{J_t}\gamma _{j,t}{\mathbb {I}}(x \in R_{j,t}) \end{aligned}$$

The gradient boosting algorithm above has two main hyperparameters: the number of iterations i.e., the number of trees constructed *T*, and the learning rate $\alpha$. We use the R package XGBoost implementation of gradient boosting [18]. The extreme gradient boosting (XGBoost) model [11] has been successfully used in many competitions and we selected it as a state-of-the-art method. We denote the default hyperparameter values as “XGBoost0”, and the optimized model as “XGBoost”. We found that the default hyperparameter values $\alpha =0.3, T=50$ work well. However, a lower learning rate $\alpha$ always resulted in a more accurate model. The optimal number of iterations *T* depends on both the predicted outcome and the learning rate. In the optimized model, we therefore set a reasonably low value to the learning rate $\alpha = 0.1$ and limited the number of iterations by early stopping based on five-fold cross-validation on training data. XGBoost is able to handle missing feature values directly by considering these as a tree splitting criterion, just like ordinary values, so there was no need to impute missing feature values first. We found that missing values gave slightly better predictions with XGBoost.

Attention should be paid to the spatial location feature here, because location may provide information that cannot be accounted for by the demographic, household or neighborhood features only. While in the STAR model location is implicitly included in the model as a spatially correlated random effect using a Markov random field term, for a tree-based model this information could be provided by simple *x* and *y* coordinates. However, this may result in orthogonal artifacts in the maps [19]. We therefore used oblique geographic coordinates (OGC) as an alternative. These are *K* additional features added to the data by a feature transformation of $x_i$ and $y_i$ coordinate features, calculated by:

$$\begin{aligned} x^{OGC_k}_i = \sqrt{x^2_i + y^2_i} \, \text {cos} \left[ \theta _k - \text {atan} \left( \frac{y_i}{x_i} \right) \right] , \end{aligned}$$

where the angle $\theta _k$ takes the values $\pi (k - 1)/K, k = 1, \dots , K$, in which *K* is a reasonably large number, chosen such that model accuracy does not improves any further. We found $K = 24$ to be a good trade-off. We added an extension “_ogc” to denote a model with this location information and “_xy” as the model with ordinary x &y-coordinates.

### Validation

To evaluate model performance, we use the data set ${\mathcal {D}}=\{(x_i,y_i)\}_{i\in {\mathcal {I}}}$ of survey respondents with known binary health-related indicators or living quality ratings. We split this data set into five mutually exclusive training and test set pairs with five-fold cross-validation, i.e., ${\mathcal {D}}_{\text {train}}\cup {\mathcal {D}}_{\text {test}}={\mathcal {D}}$ and ${\mathcal {D}}_{\text {train}}\cap {\mathcal {D}}_{\text {test}}=\emptyset$. For each pair, a model *f* is fitted to the training set ${\mathcal {D}}_{\text {train}}$ with observed responses and the unknown responses are predicted in the test set ${\mathcal {D}}_{\text {test}}$. Taking together the predictions in the five mutually exclusive tests sets, we therefore have out-of-sample predictions for the original data set ${\mathcal {D}}$.

To validate the models, two aspects are particularly important: discrimination and calibration. Discrimination only applies to classification, while calibration applies to both classification and regression. Discrimination measures to what extent the model is able to discriminate a high risk individual from a low risk individual, without necessarily considering the absolute values of the predictions. Calibration on the other hand quantifies how close the predicted probabilities or ratings are to the observed probabilities or ratings. In our classification task we aim to predict the individual probabilities as accurately as possible, so we need models that are also well calibrated.

The Receiver Operating Characteristic (ROC) is a popular discrimination visualization. The area under the ROC curve (AUC) only measures discrimination because it is calculated from the number of correct rankings of positive examples over negative ones [20]. Accuracy also considers discrimination because it is the proportion of correctly classified individuals for a given threshold value. In the case of binary responses, a calibration curve compares the predicted probability quantiles and the true probability of 1 in each quantile.

Statistics that measure both discrimination and calibration are the mean squared error (MSE) and negative log-likelihood (NLL), which we normalized by dividing by the sample size. We verified that different metrics gave consistent results across the models. We primarily report the model accuracy as the MSE, which is also a valid metric in binary classification known as the Brier score [21]:

$$\begin{aligned} \text {MSE}({\mathcal {D}}_{\text {test}}) = \frac{1}{|{\mathcal {D}}_{\text {test}}|} \sum _{i\in {\mathcal {D}}_{\text {test}}}(y_i - f(x_i))^2 \end{aligned}$$

## Results

In this section, we first investigate the prediction task in detail using the HeMo indicator “drinker”, which is the first indicator in the data set. We then summarize the results for each of the 34 health-related indicators, the 8 living quality ratings, and the 10 noise disturbance indicators.

The predicted prevalences for all indicators at neighbourhood, district and municipality level for 2020, based on the HeMo survey, can be found in the URL provided in the Online materials.

### Health-related indicator “drinker”

**Fig. 1:**
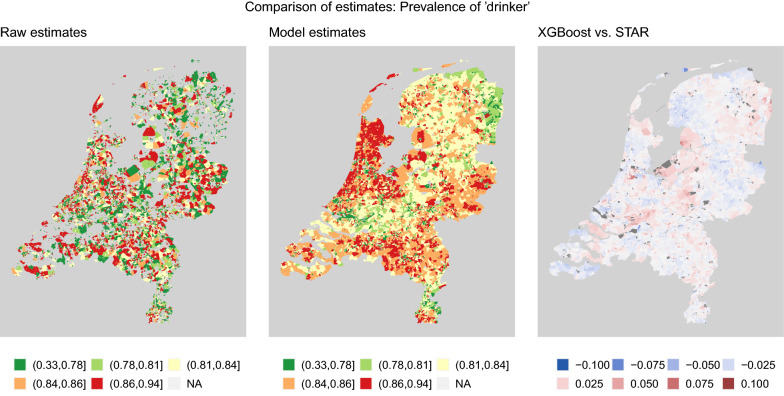
Illustration of small area estimation at neighbourhood level in the Netherlands. Prevalence of “drank alcohol in the past 12 months” based on survey responses (raw estimates), XGBoost model predictions for the population (model estimates), and the percentage point difference of two models (XGBoost vs. STAR). XGBoost is based on X and Y-coordinates

The task is to predict the binary response $y_i \in \{0,1\}$, corresponding to the health survey question “Did you drink alcohol in the past 12 months?”, given demographic and spatial characteristics of individuals. The estimated prevalences of this indicator based on XGBoost model predictions are shown in the middle panel of Fig. 1.

There are some differences in XGBoost and STAR model predictions, even though the maps they produce seem very alike. We plot the differences in predicted prevalences in the right panel of Fig. 1 and list the absolute differences in Table 4.

**Table 4:** XGBoost vs. STAR: percentage point difference in predicted prevalence

Difference in prevalence	[0,0.025)	[0.025,0.05)	[0.05,0.075)	[0.075,0.1)	[0.1,0.125)
Total neighbourhoods (%)	92.07	7.29	0.57	0.05	0.02

In Table 5 we report the accuracy, AUC, MSE, NLL, and the average time taken to train the model and predict the responses in each fold. XGBoost is the most accurate model in every metric, and it is possible to obtain predictions in the matter of seconds for XGBoost with default hyperparameters, while training the STAR model takes almost half an hour.

**Table 5:** Different accuracy metrics for “drinker” indicator

Model	Accuracy	AUC	MSE	NLL	Time
Null	0.801	0.500	0.160	0.500	**0 s**
STAR	0.814	0.737	0.138	0.437	28 min
XGBoost0_xy	0.815	0.742	0.137	0.434	6s
XGBoost_xy	**0.816**	**0.744**	**0.136**	**0.433**	3 min
XGBoost0_ogc	0.815	0.742	0.137	0.434	17s
XGBoost_ogc	**0.816**	**0.744**	**0.136**	**0.433**	6 min

Bold values indicated the best result, not statistical significance

**Fig. 2:**
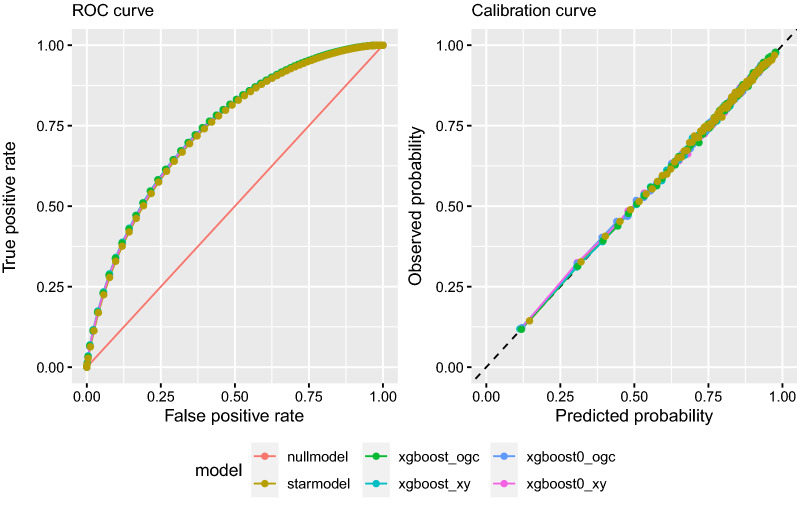
ROC and calibration curves. The ROC curve measures discrimination by plotting the false positive and true positive rates at different threshold values. The calibration curve shows how well the predicted probabilities match the true probabilities: the mean of predicted probabilities is calculated at different quantiles of true probabilities with the diagonal line indicating a perfect fit

To investigate the full profile of discrimination ability and make sure the models are well-calibrated, we calculated the ROC curve and calibration curve in Fig. 2. Since Statistics Netherlands does not allow reporting individual predictions, we used 100 different threshold values for the ROC curve and 100 different quantiles of prevalence for the calibration curve. The models appear very close to each other in discrimination ability and every model is well-calibrated.

**Fig. 3:**
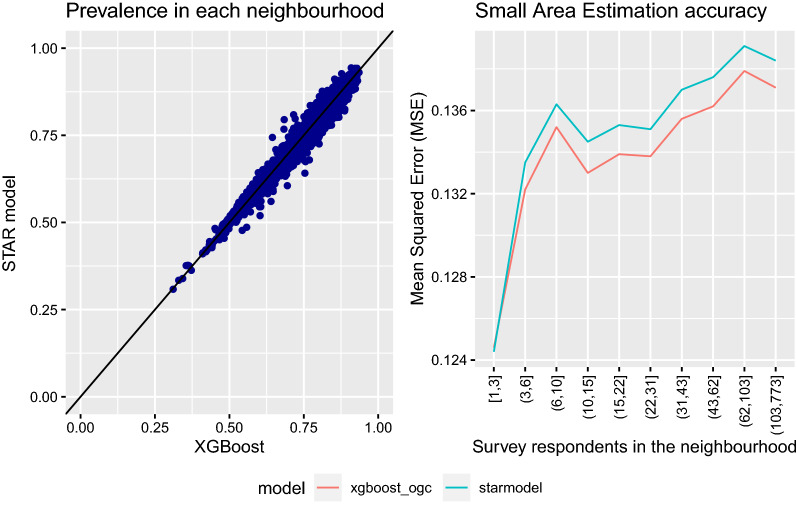
XGBoost vs. STAR predictions. We compare the predicted prevalences in each neighbourhood from the XGBoost and STAR models on a scatter plot (left). We also calculate the MSE stratified over individuals that belong to the same neighbourhood size quantile (right)

Since the primary interest is small areas, we also wanted to make sure that the new model (XGBoost) matched the previous model (STAR) in these areas. In Fig. 3, we compare the predicted prevalence in each neighborhood and measure model accuracy by MSE for different area sizes. The Pearson correlation coefficient in this example is 95%, which means that the two models predict quite similar but not identical prevalences. The XGBoost model has a lower MSE for all area sizes for the ’drinker’ indicator.

### All health-related indicators, living quality ratings, and noise disturbance

We fitted the XGBoost models (x and y, ogc) with optimized hyperparameters and the STAR model to each of the 34 health-related indicators, the 8 perceived living quality ratings, and the 10 experienced noise disturbance indicators. We measure the performance by their MSE, since the previous experiment indicated that different metrics give consistent results. Because the primary goal is to improve upon the existing statistical model by using a machine learning model, we also summarized the XGBoost vs. STAR model comparison with the following columns in each table:

- The Pearson correlation (corr) of predictions for each neighborhood.
- The % improvement in MSE of predictions (pred) obtained by XGBoost.
- The % improvement in train & prediction time (time) by XGBoost.

In Table 6 we see that XGBoost is the best model for every one of the 34 health indicators. In Table 7, we see that XGBoost is the best model for all but one of the 8 housing survey ratings. In Table 8, we find that XGBoost is always better or equally good to the STAR model for the noise disturbance indicators.

The improvements of XGBoost over STAR appear small in terms of the overall MSE, being under 1%, but there are still differences between prevalences predicted for each neighborhood as indicated by the 84–97% correlation. The training and prediction time is much improved even in the XGBoost model that searches for the optimal hyperparameters. The training and prediction times are around 10% in the health monitor, under 1% in the housing survey, and around 5% in the noise disturbance of the STAR model.

**Table 6:** Comparison of models by MSE over all health-related indicators. XGBoost oblique coordinates vs. STAR: correlation between predictions (corr), MSE reduction in percentages (pred), training and test prediction time reduction in percentages (time)

Indicator	Nullmodel	XGB_xy	XGB_ogc	STAR	Corr	Pred	Time
Drinker	0.1597	**0.1364**	**0.1364**	0.1377	0.95	0.94	78.05
Drinker_over6gd	0.0510	**0.0478**	**0.0478**	0.0480	0.93	0.42	87.55
Drinker_heavy	0.0670	**0.0642**	**0.0642**	0.0645	0.91	0.47	89.94
Drinker_excess	0.1464	**0.1402**	**0.1402**	0.1409	0.89	0.50	79.73
Drinker_excess_old	0.0623	**0.0610**	**0.0610**	0.0611	0.85	0.16	85.96
Drinker_under1gd	0.2470	0.2106	**0.2105**	0.2117	0.96	0.57	73.94
Weight_overweight	0.2493	**0.2264**	0.2265	0.2277	0.96	0.53	84.71
Weight_obese	0.1296	**0.1235**	**0.1235**	0.1240	0.94	0.40	84.80
Weight_underweight	0.0135	**0.0133**	**0.0133**	0.0133	0.90	0.00	93.04
Weight_healthly	0.2484	**0.2287**	0.2288	0.2299	0.95	0.48	85.35
Weight_overweight	0.2340	**0.2242**	**0.2242**	0.2247	0.95	0.22	92.95
Smoker	0.1133	**0.1047**	0.1048	0.1054	0.95	0.57	85.48
Smoker_past	0.2441	**0.2102**	**0.2102**	0.2110	0.98	0.38	87.72
Smoker_never	0.2467	**0.2147**	**0.2147**	0.2162	0.96	0.69	80.51
Health_reportgood	0.1785	**0.1583**	**0.1583**	0.1592	0.97	0.57	87.26
IIlness_longterm	0.2322	**0.2117**	**0.2117**	0.2126	0.97	0.42	90.56
Health_limited	0.2288	**0.1981**	**0.1981**	0.1994	0.97	0.65	88.88
Health_limited_severe	0.0508	**0.0478**	**0.0478**	0.0482	0.94	0.83	90.42
Illness_longterm_limit	0.2216	**0.1931**	0.1932	0.1945	0.97	0.67	87.97
Disability_hearing	0.0457	**0.0434**	**0.0434**	0.0435	0.93	0.23	89.39
Disability_vision	0.0495	**0.0471**	**0.0471**	0.0472	0.95	0.21	89.99
Disability_mobility	0.0985	**0.0796**	**0.0796**	0.0801	0.98	0.62	86.37
Disability_any	0.1382	**0.1153**	**0.1153**	0.1159	0.98	0.52	85.34
Feels_lifecontrol	0.0892	**0.0838**	**0.0838**	0.0842	0.95	0.48	87.39
Anxitydepression_mod	0.2415	0.2229	**0.2228**	0.2234	0.97	0.27	88.44
Anxitydepression_high	0.0442	**0.0420**	**0.0420**	0.0422	0.95	0.47	90.18
Exercise_guideline	0.2483	0.2293	**0.2292**	0.2300	0.94	0.35	80.00
Exercise_weekly	0.2488	**0.2149**	**0.2149**	0.2170	0.95	0.97	84.73
Lonely	0.2471	**0.2296**	**0.2296**	0.2306	0.96	0.43	86.80
Lonely_severe	0.0841	**0.0799**	**0.0799**	0.0802	0.95	0.37	89.13
Lonely_emotional	0.2500	**0.2304**	0.2305	0.2315	0.97	0.43	90.67
Lonely_social	0.2415	**0.2310**	0.2311	0.2317	0.94	0.26	88.60
Volunteer	0.2051	**0.1900**	**0.1900**	0.1915	0.94	0.78	83.09
Difficultyfinancial_12	0.0777	**0.0662**	**0.0662**	0.0676	0.95	2.07	86.25
Caregiver_informal	0.1318	**0.1236**	**0.1236**	0.1240	0.95	0.32	87.21
Much_stress	0.1129	**0.1045**	**0.1045**	0.1048	0.97	0.29	91.62
Severe_noise_disturb	0.0584	**0.0572**	**0.0572**	0.0574	0.95	0.35	96.10
Walk_to_work	0.1359	**0.1251**	**0.1251**	0.1257	0.95	0.48	91.47
Bike_to_work	0.1878	0.1602	**0.1601**	0.1614	0.96	0.81	79.63
Walk_or_bike_work	0.2222	0.1824	**0.1823**	0.1840	0.97	0.92	84.52

**Table 7:** Comparison of models by MSE over all living quality ratings

Indicator	Nullmodel	XGB_xy	XGB_ogc	STAR	Corr	Pred	Time
Afraid_ngbh	0.7266	0.6667	**0.6655**	0.6676	0.88	0.31	99.1
Social_cohesion	2.8648	2.5505	**2.5474**	2.5615	0.91	0.55	99.03
Satisfied_region	0.4222	0.4018	0.4001	**0.3995**	0.83	− 0.15	99.11
Annoyed_w_ngbh	0.5648	**0.5180**	0.5182	0.5203	0.88	0.4	99.24
Attached_to_ngbh	1.1237	**1.0079**	1.0082	1.0173	0.88	0.89	98.97
Satisfied_house	0.6503	**0.5386**	0.5389	0.5458	0.91	1.26	99.2
Satisfied_surroundings	0.6608	0.6056	**0.6046**	0.6054	0.89	0.13	99.01
At_home_in_ngbh	0.6382	0.6011	**0.6009**	0.6047	0.85	0.63	99.09

**Table 8:** Comparison of models by MSE over all noise disturbance indicators based on a relevant noise measurement separated by ’-’

Indicator	Nullmodel	XGB_xy	XGB_ogc	STAR	Corr	Pred	Time
Road_high-road	0.0665	**0.0635**	**0.0635**	**0.0635**	0.94	0.00	95.76
Rail_high-rail	0.0114	**0.0109**	**0.0109**	**0.0109**	0.91	0.00	96.62
Air_high-air	0.0357	0.0314	**0.0313**	**0.0313**	0.96	0.00	95.88
Road_medhigh-road	0.2234	0.2060	**0.2056**	0.2058	0.96	0.10	93.73
Rail_medhigh-rail	0.0780	0.0671	**0.0667**	0.0671	0.96	0.60	93.31
Air_medhigh-air	0.1593	0.1295	**0.1286**	**0.1286**	0.98	0.00	93.42
Road_high_gt50-road_pwrw	0.0328	**0.0320**	**0.0320**	**0.0320**	0.91	0.00	96.43
Road_medhigh_gt50-road_pwrw	0.1557	0.1465	**0.1462**	0.1467	0.95	0.34	94.11
Road_high_sm50-road_gw	0.0519	**0.0501**	**0.0501**	0.0502	0.92	0.20	96.73
Road_medhigh_sm50-road_gw	0.2045	0.1918	**0.1914**	**0.1914**	0.94	0.00	93.85
Road_high_gt50-road	0.0328	**0.0320**	**0.0320**	**0.0320**	0.90	0.00	95.94
Road_medhigh_gt50-road	0.1557	0.1472	**0.1465**	0.1467	0.93	0.14	92.50
Road_high_sm50-road	0.0519	**0.0501**	**0.0501**	**0.0501**	0.92	0.00	96.73
Road_medhigh_sm50-road	0.2045	0.1914	**0.1911**	**0.1911**	0.95	0.00	94.70

## Discussion

### Ease of application

We investigated machine learning for small area estimation using gradient boosted trees. The idea of machine learning is to use a generic learning algorithm that can be applied to any new problem. The algorithm works as a ‘black box’ model: we must only define the features as input and labels as output. An accurate model is learned as a result. This means that an explicit model specification is not required, as is the case with statistical models.

The generic learning algorithm is very flexible: it can learn non-linear effects, arbitrary interactions to several degrees, and complex spatial patterns. Spatial heterogeneity remains in the the survey data even after accounting for the features, so special attention should be paid to the spatial component. Simple x and y-coordinates work well, but oblique coordinates represent a feature transformation that may improve the accuracy. The approach allows for any possible interactions between location and the features in a flexible way, so it can also mimic Geographically Weighted Regression (GWR) if necessary.

Model application is straightforward. This model can be fitted into the entire Dutch population instead of splitting the data into subsets. The original data set can be used directly because the decision trees perform automatic feature selection, scaling, and splitting. Missing values can be used in the model instead of imputing them with complex methods. XGBoost saves a lot of time in data preprocessing, model specification, and prediction compared to the STAR model.

### Accuracy of the models

All models achieved a significant improvement over the null model in our example task, indicating that there is some relationship between the demographic and spatial features to the survey outcomes. The null model is useless in practice because it predicts the same prevalence in each area. Otherwise the models appear very close to each other in the overall metrics. The models also have a similar profile of false positive and true positive rates at different threshold values. The calibration curves show that every model is well-calibrated. Using oblique coordinates consistently resulted in lower errors than x and y-coordinates in the noise prediction task. It is possible that the signal is mostly spatial in this task and the smoothing effect benefits the model by removing orthogonal artifacts [19].

However, there are some differences if we look at the actual predictions of ‘drinker’ prevalence in each small area. Table 4 indicates that there are few neighbourhoods (0.6%) with more than $\pm 5\%$ percentage point difference in predicted prevalences, even though these can differ up to $\pm 12\%$ between XGBoost vs. STAR. Neighbourhoods with small but significant differences in the 0–5% range are more common. We could not identify characteristics of neighbourhoods that cause this difference, but spatial trends are identifiable in the right panel of Fig. 1. The STAR model has a more elaborate spatial model, while XGBoost has more flexibility in specifying how the demographic features affect the outcome. It is not possible to learn the spatial effect from very few respondents, but the spatial effect is able to compensate a biased model if many respondents are available. This bias could be caused by an oversimplified model specification or the data missing important features. For example, the study that introduced the original STAR model [4] found implausible estimates of smoking prevalence caused by the model specification missing the level of ‘education’ combined with few respondents in the neighbourhood.

These findings suggest that the overall metric may indicate little difference between the models whereas the actual prediction task shows considerable differences. It may be beneficial to pursue marginally more accurate models for better predictions into small areas. The ultimate goal is to predict prevalences accurately at neighborhood level, and we hope that improvement in accuracy metrics works as a surrogate measure of this ability. The overall metric is no guarantee that the model is better for small areas. For this reason, we verified that XGBoost was uniformly better for all area sizes. The smallest difference was observed for very small areas.

### Interpretation of model fits

**Fig. 4:**
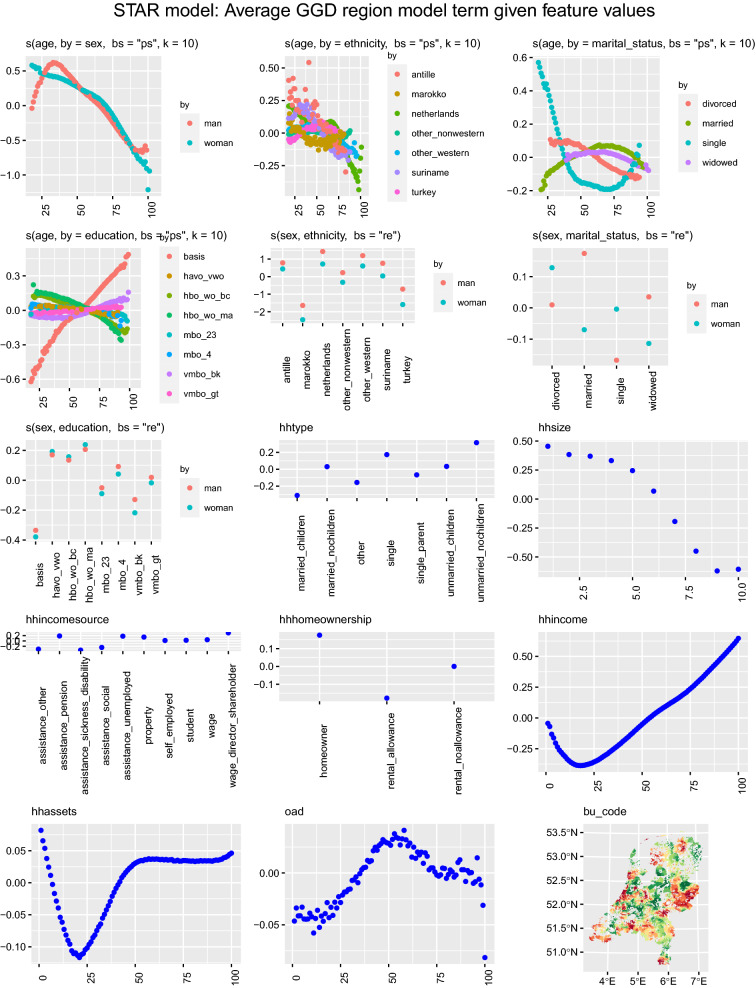
STAR model terms for “drinker” indicator.. Because a different model is fitted for each of the 25 GGD regions, we calculate the average term value over these models for a given feature value. Even though interpretation is seen as a strength of statistical models, it is quite non-trivial to interpret such a complex model. Compared to the XGBoost SHAP values below, the STAR model appears to have a similar interpretation

In many cases it is of interest to interpret the predictive model. The STAR model is directly interpretable: each of the terms describes how the prediction changes as a function of one feature value when all other features are held constant. It is possible to calculate both effect sizes and statistical significance from this model. In Fig. 4 in the Appendix, we plot all the different terms included in the model. Even though the model is interpretable in theory, it is quite challenging to understand the effect of several features (age, sex, ethnicity, marital_status, education) since this model includes so many interactions.

**Fig. 5:**
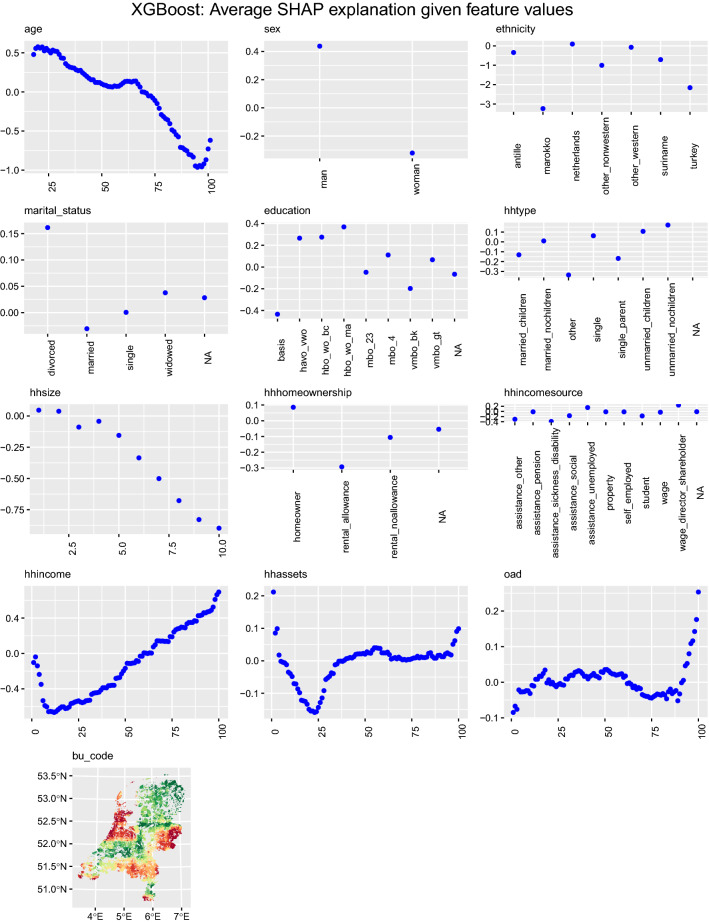
XGBoost SHAP values for “drinker” indicator.. Because SHAP values explain each individual’s prediction as a sum of the contribution of their features, we calculate the average SHAP value of these individuals for a given feature value. These have intuitive interpretations. Positive contributions to drinking are: age in early 20s, sex is man, being divorced, higher socioeconomic status. Negative contributions: being retired, sex is woman, ethnic backgrounds where Islam is the main religion, larger household size, income and assets around the lowest 25%

Shapley additive explanations [22] is one approach to the problem of interpreting machine learning models. A Shapley value is calculated for every feature value of every individual. On a conceptual level, the SHAP values explain every individual’s prediction as a sum of contributions of their features. In Fig. 5 in the Appendix, we plot the mean SHAP value of XGBoost at every feature value. They provide an interpretation which is very similar to the STAR model.

### Limitations and possible extensions

Many other surveys that allow the linking of individual’s survey data to their administrative data could be investigated with the same method. The main limitation in our approach is the amount and quality of data. Regression based SAE requires an administrative data set for the population and a survey data set of reasonable size for a subset of the population.

The Netherlands collects a high quality administrative data set which is available in a secured CBS environment, but this is not the case in many other countries. Many features were available to train a complex model based on machine learning, but with less information simpler models could be competitive. Another limitation may be the survey size. We had tens to hundreds of thousands of respondents in each survey. With considerably fewer respondents it is possible that simpler approaches are sufficient because it is not possible to learn a complex model from little data. We have not investigated the minimum number of respondents required, but this may be a topic for further research.

Our surveys were a stratified sample throughout the Netherlands [1, 2], and the estimation procedure can in principle account for a non-representative sample. This is because a regression approach assumes data of the entire population is available and predicts the answers of individuals who are not in the survey. For example, if high income individuals answer the survey more, directly using the survey would give biased results. The model based estimates should still be correct, because we predict the answers for both high and low income individuals as they are represented in the population. The only requirement is that the model is unbiased for the response, given the individual’s features. Simpler methods like survey weighting take this into account by incorporating design weights and post-stratification.

## Conclusion

We have used gradient boosted decision trees, as implemented in the ‘XGBoost’ R package, as a machine learning method for providing small area estimates of public health, housing and well-being in the population of Netherlands. The labels are responses in a survey and the features come from a registry data set of demographic and spatial variables. These responses are available for a small subset of the population, but the registry data is available for the entire adult population of Netherlands. The missing survey responses can therefore be predicted by a model trained on the observed responses, and these predictions aggregated into the predicted prevalence or average rating in each small area.

We have seen that machine learning has multiple benefits. A single machine learning method can learn the prediction task in a matter of minutes with similar accuracy as purpose built models for small area estimation in the Netherlands. Gradient boosted decision trees are able to slightly improve the accuracy, and drastically improve the training and prediction time. Default hyperparameters worked well and tuning achieved only a small performance improvement. The model is unmatched in the simplicity and ease of use. The statistician does not have to do a complex, time consuming, and error-prone model specification process. The method automatically learns non-linear feature effects, interactions to many degrees, and complex spatial signal from x and y-coordinates. Accuracy and interpretation could be further improved using the oblique coordinate transformation when the signal was mostly spatial. These results suggest that machine learning is an attractive alternative for small area estimation.

## Data Availability

Tables https://statline.rivm.nl/#/RIVM/nl/dataset/50090NED. Code https://gitlab.com/majuvi/smap-2020-paper. Data can be accessed in the CBS remote access environment by authorized researchers, see https://www.cbs.nl/en-gb/onze-diensten/customised-services-microdata/microdata-conducting-your-own-research.

## Appendix

**Table 9:** Subset of health-related indicators from the Public health monitor (‘HeMo’)

Health-related indicator	Original name	Description
Drinker	lfala217	Drank alcohol in the past 12 months
Drinker_over6gd	lfalaa213	Drinks over 6 glasses of alcohol per day
Drinker_heavy	lfala213	Drinks at least 1 times a week at least 6 (M) or 4 (F) glasses per day
Drinker_excess	lfals231	Drinks alcohol in excess of recommendation
Drinker_excess_old	lfals230	Drinks alcohol in excess of recommendation (old definition)
Drinker_under1gd	lfals232	Drinks under 1 glass of alcohol per day
Weight_overweight	aggws204	Overweight, BMI 25 or higher
Weight_obese	aggws205	Obese, BMI 30 or higher
Weight_underweight	aggws206	Underweight, BMI 18.5 or lower
Weight_healthy	aggws207	Healthy weight, BMI 18.5–25
Weight_overweight_moderate	aggws208	Moderately overweight, BMI 25–30
Smoker	lfrka205	Smoker
Smoker_past	lfrka206	Smoked in the past
Smoker_never	lfrka207	Has never smoked
Health_reportgood	klgga208	Considers own health as good
Illness_longterm	calga260	Has an illness of duration 6 months or longer
Health_limited	calga264	Is limited in daily life by problems with health
Health_limited_severe	calga265	Is seriously limited in daily life by problems with health
Illness_longterm_limited	calga267	Is limited in daily life by problems with health 6 months or longer
Disability_hearing	lgbps203	Has a hearing disability (great difficulty with 1 of 2 OECD items)
Disability_vision	lgbps204	Has a vision disability (great difficulty with 1 of 2 OECD items)
Disability_mobility	lgbps205	Has a mobility disability (great difficulty with 1 of 3 OECD items)
Disability_any	lgbps209	Has a hearing, vision, or mobility disability (1 of 7 OECD items)
Feels_lifecontrol	ggrls203	Feels moderate or much control over their own life
Anxietydepression_moderate	ggada202	Moderate or high risk of anxiety disorder or depression
Anxietydepression_high	ggada203	High risk of anxiety disorder or depression
Exercise_guideline	ki_rlbew2017	Complies with the 2017 exercise guideline
Exercise	kisporter	Core indicator of actual exercise
Lonely	ggees217	Is lonely
Lonely_severe	ggees209	Is seriously lonely
Lonely_emotional	ggees218	Is emotionally lonely
Lonely_social	ggees219	Is socially lonely
Volunteer	mmvwa201	Does volunteer work
Difficultyfinancial_12m	mmika201	In the past 12 months experienced difficulty with household income
Caregiver_informal	mcmzgs203	Caregiver (at least 3 months and/or at least 8 hours a week)
Much_stress	ggsts16	Experienced a lot of stress in the past 4 weeks
Severe_noise_disturb	woghba218	Experiences severe noise disturbance by neighbors
Walk_to_work	wwlmwk	(Partly) walks to work at least 1 day a week
Bike_to_work	wwfmwk	(Partly) bikes to work at least 1 day a week
Walk_or_bike_to_work	wwfmwk, wwlmwk	(Partly) bikes or walks to work at least 1 day a week

**Table 10:** Perceived living quality ratings from the Housing survey (‘WoON’)

Living quality rating	Original name	Description
Social cohesion	Cohesie	GSB indicator of social quality
Satisfaction house	Twoning	Satisfaction with the current home
Satisfaction surroundings	Twoonomg	Satisfaction with the surroundings
Satisfaction region	Tevrstr	Satisfaction with the region where you live
Bothered by neighborhood	Tvervele	It’s annoying to live in this neighborhood
At home in neighborhood	Brtthuis	I feel at home in this neighborhood
Afraid in neighborhood	Brtveilig	Afraid of being harassed or robbed in this neighborhood

**Table 11:** Experienced noise disturbance subset of the Public health monitor (’Noise’)

Noise disturbance indicator	Original name	Description
Road_medhigh_gt50	WOGHBA202	Moderate or serious noise nuisance from road traffic $> 50 \,\hbox {km/h}$
Road_high_gt50	WOGHBA203	Serious noise nuisance from road traffic $> 50 \,\hbox {km/h}$
Road_medhigh_sm50	WOGHBA205	Moderate or serious noise nuisance from road traffic $< 50 \,\hbox {km/h}$
Road_high_sm50	WOGHBA206	Serious noise nuisance from road traffic $< 50 \,\hbox {km/h}$
Road_medhigh	WOGHBA202/205	Moderate or serious noise nuisance from road traffic
Road_high	WOGHBA203/206	Serious noise nuisance from road traffic
Rail_medhigh	WOGHBA208	Moderate or serious noise nuisance from train traffic
Rail_high	WOGHBA209	Serious noise nuisance from train traffic
Ail_medhigh	WOGHBA211	Moderate or serious noise nuisance from air traffic
Ail_high	WOGHBA212	Serious noise nuisance from air traffic

**Table 12:** Estimated noise level (dB) from the RIVM noise dispersion model

Estimated noise level	Original name	Description
Air	lden_air	Noise from air traffic
Rail	lden_rail	Noise from railway traffic
Road	lden_wegv	Noise from road traffic
Road_gw	lden_wegv_gw	Noise from municipal roads
Road_pwrw	lden_wegv_pw, lden_wegv_rw	Noise from secondary and main motorways

**Table 13:** Description of Dutch education levels

Education level	Description
Basis	Primary education
VMBObk	Lower secondary education (predominantly practical)
VMBOgt	Lower secondary education (predominantly theoretical)
MBO23	Post-secondary vocational education (lower levels)
MBO4	Post-secondary vocational education (highest level)
HAVO-VWO	Higher secondary education
HBO-WO-BAC	Undergraduate degree
HBO-WO-M/PhD	Graduate/doctoral degree
